# Supplementary material for: Early Alterations in Structural and Functional Properties in the Neuromuscular Junctions of Mutant FUS Mice
Source: Int J Mol Sci. 2023 May 19;24(10):9022. doi: 10.3390/ijms24109022 (PMC10218837; doi:10.3390/ijms24109022)
Supplement: Supplementary file 1 [file ijms-24-09022-s001.zip › ijms-2355629-supplementary.pdf]

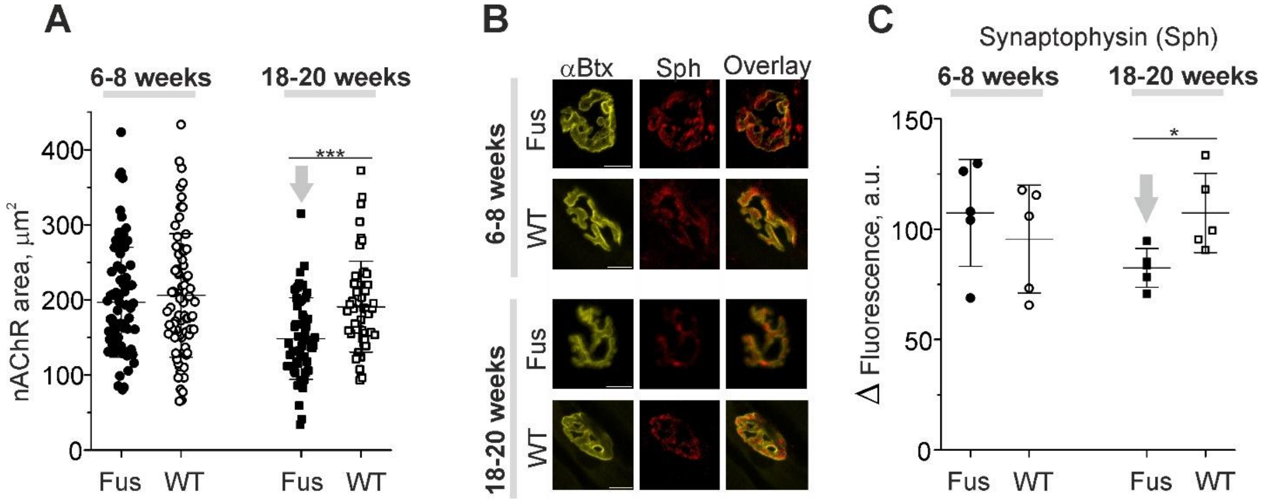

Supplementary Figure S1. A-Measurements of nAChR area (in µm^2), assessed as α-Btx positive regions. Shown the values of individual NMJs from 6 mice *per* group. ***P<0.001 by Kolmogorov-Smirnov test between NMJs from FUS and WT animals. B – typical fluorescent images of NMJs labeled with αBtx and anti-synaptophysin (Sph) antibody. Scale bars – 20 µm. C – Quantification of synaptophysin fluorescence in motor nerve terminals. n=5 mice per group (13-15 NMJs from each muscle). *P<0.05 by Mann-Whitney U test compared to WT. 6-8-week-old and 18-20-week-old mice with ΔFUS(1-359) represent animals at pre-onset and onset stages.

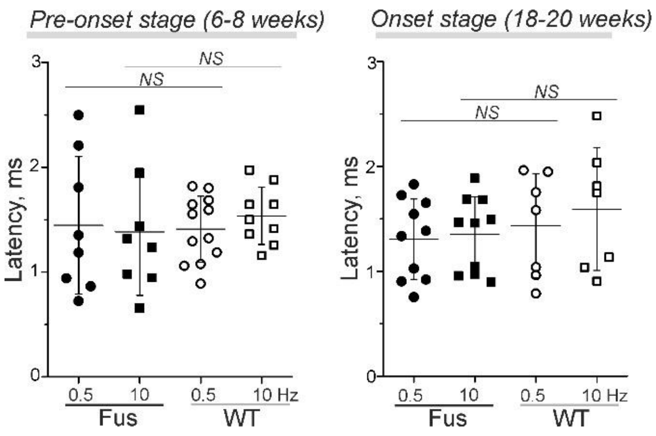

Supplementary Figure S2. Quantification of latency (interval from stimulus artefact to presynaptic action potential) in the motor axon of ΔFUS(1-359) mice *vs* WT mice. Shown comparison of the latency at 0.5 and 10 Hz nerve stimulation in ΔFUS(1-359) mice at pre-onset and onset stages as compared to age-matched WT animals. n=8-12 muscles from individual mice *per* group; 300 action potentials were analyzed in each muscle.

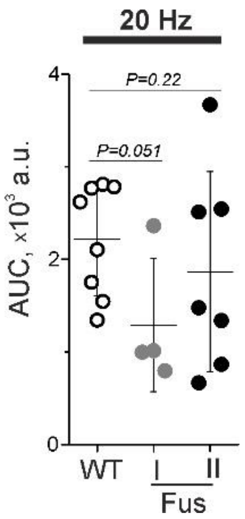

Supplementary Figure S3. Area under curve of intraterminal Ca^{2+} elevation upon one-minute stimulation at 20 Hz. Shown data from ΔFUS(1-359) mice at pre-onset (I; 6-8 week-old) and onset (II; 18-20 week-old) stages as compared to WT (6-8 + 18-20 week-old) mice. Nerve terminal $[Ca^{2+}]_{in}$ dynamics are shown in Fig. 7B. n=4-8 muscles from individual mice per group (WT: 36 NMJs of 8 mice; FUS I: 18 NMJ of 4 mice; 22 NMJs of 7 mice). $P_{asymp.}$=0.051 (P_{exact}=0.049), $P_{asymp.}$= 0.224 by Mann-Whitney *U* test.
